# Supplementary material for: Diminished responses to bodily threat and blunted interoception in suicide attempters
Source: eLife. 2020 Apr 7;9:e51593. doi: 10.7554/eLife.51593 (PMC7138608; doi:10.7554/eLife.51593)
Supplement: Source code 1. [file elife-51593-code1.zip › eLife_analyses_rev1.html]

Interoception & Suicide: Primary Analyses, Results, and Figures


# Interoception & Suicide: Primary Analyses, Results, and Figures

### load libraries and data

```
library(dplyr)
library(tidyr)
library(lme4)
library(lmerTest)
library(ggplot2)
library(tableone)
library(rcompanion)
library(GGally)
library(sjPlot)
library(pbkrtest)
library(r2glmm)
library(ggcorrplot)


lm.beta.lmer <- function(mod) {
   b <- fixef(mod)[-1]
   sd.x <- apply(getME(mod,"X")[,-1],2,sd)
   sd.y <- sd(getME(mod,"y"))
   b*sd.x/sd.y
}

my.data <- read.csv('data_t1k_suicide.csv')
```

---

## Analaysis of Breath Hold Data

#### Results reported on page 12 of manuscript and depicted in Figure 1

---

#### Duration analysis

```
duration = my.data %>% dplyr::select ("id", "Group", "bh_hold_duration_0", "bh_hold_duration_2")

duration <- gather(duration,
                   value = "value",
                   key = "trial",
                   bh_hold_duration_0, bh_hold_duration_2)

duration_lmer <- lmer(value ~ Group*trial + (1|id), data = duration, na.action = na.exclude)
anova(duration_lmer, ddf="Kenward-Roger", type = "marginal")
```

```
## Marginal Analysis of Variance Table with Kenward-Roger's method
##              Sum Sq Mean Sq NumDF   DenDF F value   Pr(>F)    
## Group        491.84  491.84     1 121.837  4.4778  0.03637 *  
## trial       2216.18 2216.18     1  97.011 20.1765 1.95e-05 ***
## Group:trial   29.14   29.14     1  97.402  0.2653  0.60767    
## ---
## Signif. codes:  0 '***' 0.001 '**' 0.01 '*' 0.05 '.' 0.1 ' ' 1
```

```
summary(duration_lmer, ddf="Kenward-Roger")
```

```
## Linear mixed model fit by REML. t-tests use Kenward-Roger's method [
## lmerModLmerTest]
## Formula: value ~ Group * trial + (1 | id)
##    Data: duration
## 
## REML criterion at convergence: 1695
## 
## Scaled residuals: 
##     Min      1Q  Median      3Q     Max 
## -3.8239 -0.4128 -0.0355  0.3533  3.7541 
## 
## Random effects:
##  Groups   Name        Variance Std.Dev.
##  id       (Intercept) 416.0    20.40   
##  Residual             109.8    10.48   
## Number of obs: 199, groups:  id, 100
## 
## Fixed effects:
##                                    Estimate Std. Error      df t value Pr(>|t|)
## (Intercept)                          42.978      2.781 120.322  15.456  < 2e-16
## GroupYesSA                           10.445      4.936 121.837   2.116   0.0364
## trialbh_hold_duration_2               8.074      1.797  97.011   4.492 1.95e-05
## GroupYesSA:trialbh_hold_duration_2   -1.653      3.209  97.402  -0.515   0.6077
##                                       
## (Intercept)                        ***
## GroupYesSA                         *  
## trialbh_hold_duration_2            ***
## GroupYesSA:trialbh_hold_duration_2    
## ---
## Signif. codes:  0 '***' 0.001 '**' 0.01 '*' 0.05 '.' 0.1 ' ' 1
## 
## Correlation of Fixed Effects:
##             (Intr) GrpYSA tr___2
## GroupYesSA  -0.563              
## trlbh_hl__2 -0.323  0.182       
## GrpYSA:___2  0.181 -0.331 -0.560
```

```
sjPlot::tab_model(duration_lmer, show.std = TRUE, show.se=TRUE, show.df = TRUE, show.stat = TRUE)
```

|  | value | | | | | | | |
| --- | --- | --- | --- | --- | --- | --- | --- | --- |
| Predictors | Estimates | std. Error | std. Beta | CI | standardized CI | Statistic | p | df |
| (Intercept) | 42.98 | 2.78 | -0.30 | 37.53 – 48.43 | -0.53 – -0.07 | -2.52 | 0.01 | 120.30 |
| Group [YesSA] | 10.44 | 4.94 | 0.44 | 0.77 – 20.12 | 0.03 – 0.86 | 2.12 | 0.03 | 121.81 |
| trial [bh\_hold\_duration\_2] | 8.07 | 1.80 | 0.34 | 4.55 – 11.60 | 0.19 – 0.49 | 4.49 | 0.00 | 96.98 |
| Group [YesSA] \* trial [bh\_hold\_duration\_2] | -1.65 | 3.21 | -0.07 | -7.94 – 4.64 | -0.34 – 0.20 | -0.52 | 0.61 | 97.38 |
| Random Effects | | | | | | | | |
| σ2 | 109.84 | | | | | | | || τ00 id | 415.97 | | | | | | | || ICC | 0.79 | | | | | | | || N id | 100 | | | | | | | || Observations | 199 | | | | | | | |
| Marginal R2 / Conditional R2 | 0.062 / 0.804 | | | | | | | |

```
r2beta(duration_lmer, method = 'kr')
```

```
##        Effect   Rsq upper.CL lower.CL
## 1       Model 0.186    0.318    0.094
## 3       trial 0.173    0.316    0.062
## 2       Group 0.042    0.147    0.001
## 4 Group:trial 0.003    0.062    0.000
```

---

#### Oxygen concentration analysis

```
o2 = my.data %>% dplyr::select ("id", "Group", "bh_post_o2_0_calibrated", "bh_post_o2_2_calibrated")
o2 <- gather(o2,
                   value = "value",
                   key = "trial",
                   bh_post_o2_0_calibrated, bh_post_o2_2_calibrated)

o2_lmer <- lmer(value ~ Group*trial + (1|id), data = o2, na.action = na.exclude)
anova(o2_lmer, ddf="Kenward-Roger", type = "marginal")
```

```
## Marginal Analysis of Variance Table with Kenward-Roger's method
##             Sum Sq Mean Sq NumDF   DenDF F value  Pr(>F)  
## Group       4.8400  4.8400     1 132.268  5.0045 0.02696 *
## trial       5.9538  5.9538     1  91.086  6.1561 0.01493 *
## Group:trial 1.4025  1.4025     1  93.312  1.4501 0.23155  
## ---
## Signif. codes:  0 '***' 0.001 '**' 0.01 '*' 0.05 '.' 0.1 ' ' 1
```

```
summary(o2_lmer, ddf="Kenward-Roger")
```

```
## Linear mixed model fit by REML. t-tests use Kenward-Roger's method [
## lmerModLmerTest]
## Formula: value ~ Group * trial + (1 | id)
##    Data: o2
## 
## REML criterion at convergence: 680.8
## 
## Scaled residuals: 
##     Min      1Q  Median      3Q     Max 
## -2.7151 -0.4575  0.0059  0.3992  3.5281 
## 
## Random effects:
##  Groups   Name        Variance Std.Dev.
##  id       (Intercept) 1.8641   1.3653  
##  Residual             0.9671   0.9834  
## Number of obs: 189, groups:  id, 96
## 
## Fixed effects:
##                                         Estimate Std. Error       df t value
## (Intercept)                              13.2710     0.2040 129.9607  65.039
## GroupYesSA                               -0.8516     0.3807 132.2679  -2.237
## trialbh_post_o2_2_calibrated             -0.4185     0.1687  91.0864  -2.481
## GroupYesSA:trialbh_post_o2_2_calibrated   0.3890     0.3231  93.3120   1.204
##                                         Pr(>|t|)    
## (Intercept)                               <2e-16 ***
## GroupYesSA                                0.0270 *  
## trialbh_post_o2_2_calibrated              0.0149 *  
## GroupYesSA:trialbh_post_o2_2_calibrated   0.2316    
## ---
## Signif. codes:  0 '***' 0.001 '**' 0.01 '*' 0.05 '.' 0.1 ' ' 1
## 
## Correlation of Fixed Effects:
##             (Intr) GrpYSA t__2_2
## GroupYesSA  -0.536              
## trlbh__2_2_ -0.413  0.222       
## GYSA:__2_2_  0.216 -0.415 -0.522
```

```
sjPlot::tab_model(o2_lmer, show.std = TRUE, show.se=TRUE, show.stat = TRUE)
```

|  | value | | | | | | |
| --- | --- | --- | --- | --- | --- | --- | --- |
| Predictors | Estimates | std. Error | std. Beta | CI | standardized CI | Statistic | p |
| (Intercept) | 13.27 | 0.20 | 0.23 | 12.87 – 13.67 | -0.00 – 0.46 | 1.92 | 0.05 |
| Group [YesSA] | -0.85 | 0.38 | -0.50 | -1.60 – -0.11 | -0.93 – -0.06 | -2.24 | 0.03 |
| trial [bh\_post\_o2\_2\_calibrated] | -0.42 | 0.17 | -0.24 | -0.75 – -0.09 | -0.44 – -0.05 | -2.48 | 0.01 |
| Group [YesSA] \* trial [bh\_post\_o2\_2\_calibrated] | 0.39 | 0.32 | 0.23 | -0.24 – 1.02 | -0.14 – 0.60 | 1.20 | 0.23 |
| Random Effects | | | | | | | |
| σ2 | 0.97 | | | | | | || τ00 id | 1.86 | | | | | | || ICC | 0.66 | | | | | | || N id | 96 | | | | | | || Observations | 189 | | | | | | |
| Marginal R2 / Conditional R2 | 0.040 / 0.672 | | | | | | |

```
r2beta(o2_lmer, method = "kr")
```

```
##        Effect   Rsq upper.CL lower.CL
## 1       Model 0.072    0.191     0.02
## 2       Group 0.036    0.141     0.00
## 3       trial 0.020    0.111     0.00
## 4 Group:trial 0.015    0.100     0.00
```

---

#### Carbon dioxide concentration analysis

```
co2 = my.data %>% dplyr::select ("id", "Group", "bh_post_co2_0_calibrated", "bh_post_co2_2_calibrated")
co2 <- gather(co2,
                   value = "value",
                   key = "trial",
                   bh_post_co2_0_calibrated, bh_post_co2_2_calibrated)

co2_lmer <- lmer(value ~ Group*trial + (1|id), data = co2, na.action = na.exclude)
anova(co2_lmer, ddf="Kenward-Roger", type = "marginal")
```

```
## Marginal Analysis of Variance Table with Kenward-Roger's method
##              Sum Sq Mean Sq NumDF   DenDF F value  Pr(>F)  
## Group       0.59494 0.59494     1 120.325  5.5207 0.02042 *
## trial       0.06053 0.06053     1  91.043  0.5617 0.45552  
## Group:trial 0.08182 0.08182     1  92.708  0.7593 0.38581  
## ---
## Signif. codes:  0 '***' 0.001 '**' 0.01 '*' 0.05 '.' 0.1 ' ' 1
```

```
summary(co2_lmer, ddf="Kenward-Roger")
```

```
## Linear mixed model fit by REML. t-tests use Kenward-Roger's method [
## lmerModLmerTest]
## Formula: value ~ Group * trial + (1 | id)
##    Data: co2
## 
## REML criterion at convergence: 313.3
## 
## Scaled residuals: 
##     Min      1Q  Median      3Q     Max 
## -5.0155 -0.3411  0.0287  0.3778  3.6696 
## 
## Random effects:
##  Groups   Name        Variance Std.Dev.
##  id       (Intercept) 0.3404   0.5835  
##  Residual             0.1078   0.3283  
## Number of obs: 189, groups:  id, 96
## 
## Fixed effects:
##                                           Estimate Std. Error        df t value
## (Intercept)                                5.81792    0.08119 118.41293  71.662
## GroupYesSA                                 0.35519    0.15117 120.32513   2.350
## trialbh_post_co2_2_calibrated              0.04219    0.05630  91.04275   0.749
## GroupYesSA:trialbh_post_co2_2_calibrated  -0.09416    0.10806  92.70778  -0.871
##                                          Pr(>|t|)    
## (Intercept)                                <2e-16 ***
## GroupYesSA                                 0.0204 *  
## trialbh_post_co2_2_calibrated              0.4555    
## GroupYesSA:trialbh_post_co2_2_calibrated   0.3858    
## ---
## Signif. codes:  0 '***' 0.001 '**' 0.01 '*' 0.05 '.' 0.1 ' ' 1
## 
## Correlation of Fixed Effects:
##             (Intr) GrpYSA t__2_2
## GroupYesSA  -0.537              
## trlbh__2_2_ -0.347  0.186       
## GYSA:__2_2_  0.181 -0.349 -0.521
```

```
sjPlot::tab_model(co2_lmer, show.std = TRUE, show.se=TRUE, show.stat = TRUE)
```

|  | value | | | | | | |
| --- | --- | --- | --- | --- | --- | --- | --- |
| Predictors | Estimates | std. Error | std. Beta | CI | standardized CI | Statistic | p |
| (Intercept) | 5.82 | 0.08 | -0.15 | 5.66 – 5.98 | -0.39 – 0.08 | -1.28 | 0.20 |
| Group [YesSA] | 0.36 | 0.15 | 0.52 | 0.06 – 0.65 | 0.09 – 0.96 | 2.35 | 0.02 |
| trial [bh\_post\_co2\_2\_calibrated] | 0.04 | 0.06 | 0.06 | -0.07 – 0.15 | -0.10 – 0.22 | 0.75 | 0.45 |
| Group [YesSA] \* trial [bh\_post\_co2\_2\_calibrated] | -0.09 | 0.11 | -0.14 | -0.31 – 0.12 | -0.45 – 0.17 | -0.87 | 0.38 |
| Random Effects | | | | | | | |
| σ2 | 0.11 | | | | | | || τ00 id | 0.34 | | | | | | || ICC | 0.76 | | | | | | || N id | 96 | | | | | | || Observations | 189 | | | | | | |
| Marginal R2 / Conditional R2 | 0.042 / 0.770 | | | | | | |

```
r2beta(co2_lmer, method = "kr")
```

```
##        Effect   Rsq upper.CL lower.CL
## 2       Group 0.047    0.159    0.001
## 1       Model 0.043    0.151    0.009
## 4 Group:trial 0.008    0.082    0.000
## 3       trial 0.000    0.053    0.000
```

---

#### Benjamini correction

```
#Benjamini correction for group contrast across duration, co2, and o2
bh.p = c(0.0364,0.027,0.020)
p.adjust(bh.p, method = "BH")
```

```
## [1] 0.0364 0.0364 0.0364
```

---

### Plots of breath hold results (Figure 1)

```
my.data %>% 
   mutate(Group = factor(Group, 
            levels=c("YesSA","NoSA"))) %>% 
  ggplot() + theme_classic() +
  aes(x=Group,y=mean_hold_duration, fill=Group) +  
  geom_dotplot(binaxis='y', stackdir='center', alpha=.99) +
  geom_crossbar(stat="summary", fun.y=mean, fun.ymax=mean, fun.ymin=mean, fatten=2, width=.4) +
  scale_fill_manual(values=c("#3182bd","#deebf7")) +
  scale_x_discrete(" ",
                   labels=c("Attempters", "Non-Attempters")) +
  scale_y_continuous("Breath-Hold Duration (seconds)", breaks=c(0,30,60,90,120)) +
  theme(axis.title.y = element_text(size=14),
        axis.text.y = element_text(size=14),
        axis.text.x = element_text(size=14))
```

```
my.data %>% 
  mutate(Group = factor(Group, 
    levels=c("YesSA","NoSA"))) %>% 
  ggplot() + theme_classic() +
  aes(x=Group, y=mean_post_o2) +
  stat_summary(fun.y = mean, geom="point", position ="dodge", color = "Black", width = .7) +
  stat_summary(fun.data = mean_se, geom = "errorbar", width=.2, position=position_dodge(.7)) +
  scale_x_discrete(" ",
                   labels=c("Attempters", "Non-Attempters")) +
  scale_y_continuous("Exhaled O2 (%)",breaks=c(8.0,8.3,8.6,8.9)) +
  ggtitle(" ") +
  coord_cartesian(ylim=c(7.9,9.1)) +
  theme(axis.title.y = element_text(size=14),
        axis.text.y = element_text(size=14),
        axis.text.x = element_text(size=14))
```

```
my.data %>% 
  mutate(Group = factor(Group, 
    levels=c("YesSA","NoSA"))) %>% 
  ggplot() + theme_classic() +
  aes(x=Group, y=mean_post_co2) +
  stat_summary(fun.y = mean, geom="point", position ="dodge", color = "Black", width = .7) +
  stat_summary(fun.data = mean_se, geom = "errorbar", width=.2, position=position_dodge(.7)) +
  scale_x_discrete(" ",
                   labels=c("Attempters", "Non-Attempters")) +
  scale_y_continuous("Exhaled CO2 (%)", breaks=c(5.8,6.0,6.2,6.4)) +
  ggtitle(" ") +
  coord_cartesian(ylim=c(5.7,6.5)) +
  theme(axis.title.y = element_text(size=14),
        axis.text.y = element_text(size=14),
        axis.text.x = element_text(size=14))
```

---

## Analysis for Cold Pressor Challenge

#### Results reported in pg 13 of manuscript, depicted in Figure 2

---

```
cp = my.data %>% dplyr::select ("id",
                                "Group", 
                                "cp_time_to_mild_pain_corrected",
                                "cp_time_to_moderate_pain_corrected", 
                                "cp_time_to_peak_pain_corrected", 
                                "cp_time_hand_in_water")
cp$mild = cp$cp_time_to_mild_pain_corrected
cp$moderate = cp$cp_time_to_moderate_pain_corrected
cp$peak = cp$cp_time_to_peak_pain_corrected
cp$removal = cp$cp_time_hand_in_water

cp <- gather(cp,
              value = "value",
              key = "timepoint",
              mild, moderate, peak, removal)

cp_mod <- lmer(value ~ timepoint*Group + (1|id), data = cp, na.action = na.exclude)
anova(cp_mod, ddf="Kenward-Roger", type = "marginal")
```

```
## Marginal Analysis of Variance Table with Kenward-Roger's method
##                 Sum Sq Mean Sq NumDF  DenDF F value  Pr(>F)    
## timepoint        88400 29466.7     3 276.16 86.7841 < 2e-16 ***
## Group                3     3.3     1 229.98  0.0096 0.92212    
## timepoint:Group   2947   982.3     3 277.36  2.8930 0.03573 *  
## ---
## Signif. codes:  0 '***' 0.001 '**' 0.01 '*' 0.05 '.' 0.1 ' ' 1
```

```
summary(cp_mod, ddf="Kenward-Roger")
```

```
## Linear mixed model fit by REML. t-tests use Kenward-Roger's method [
## lmerModLmerTest]
## Formula: value ~ timepoint * Group + (1 | id)
##    Data: cp
## 
## REML criterion at convergence: 3364.7
## 
## Scaled residuals: 
##     Min      1Q  Median      3Q     Max 
## -1.8383 -0.5804 -0.0139  0.4187  3.5176 
## 
## Random effects:
##  Groups   Name        Variance Std.Dev.
##  id       (Intercept) 289.9    17.03   
##  Residual             339.5    18.43   
## Number of obs: 377, groups:  id, 95
## 
## Fixed effects:
##                              Estimate Std. Error       df t value Pr(>|t|)    
## (Intercept)                   11.4928     3.0423 225.9036   3.778 0.000203 ***
## timepointmoderate              7.6466     3.1601 276.1631   2.420 0.016179 *  
## timepointpeak                 24.9733     3.1601 276.1631   7.903 6.54e-14 ***
## timepointremoval              46.8835     3.1601 276.1631  14.836  < 2e-16 ***
## GroupYesSA                     0.5646     5.7687 229.9839   0.098 0.922117    
## timepointmoderate:GroupYesSA   3.4737     6.0087 276.1631   0.578 0.563659    
## timepointpeak:GroupYesSA       6.2426     6.0087 276.1631   1.039 0.299753    
## timepointremoval:GroupYesSA   16.6877     5.9874 278.5632   2.787 0.005683 ** 
## ---
## Signif. codes:  0 '***' 0.001 '**' 0.01 '*' 0.05 '.' 0.1 ' ' 1
## 
## Correlation of Fixed Effects:
##             (Intr) tmpntm tmpntp tmpntr GrpYSA tmpntm:GYSA tmpntp:GYSA
## timepntmdrt -0.519                                                    
## timepointpk -0.519  0.500                                             
## timepntrmvl -0.519  0.500  0.500                                      
## GroupYesSA  -0.527  0.274  0.274  0.274                               
## tmpntm:GYSA  0.273 -0.526 -0.263 -0.263 -0.521                        
## tmpntp:GYSA  0.273 -0.263 -0.526 -0.263 -0.521  0.500                 
## tmpntr:GYSA  0.274 -0.264 -0.264 -0.528 -0.529  0.502       0.502
```

```
sjPlot::tab_model(cp_mod, show.std = TRUE, show.se=TRUE, show.stat = TRUE)
```

|  | value | | | | | | |
| --- | --- | --- | --- | --- | --- | --- | --- |
| Predictors | Estimates | std. Error | std. Beta | CI | standardized CI | Statistic | p |
| (Intercept) | 11.49 | 3.04 | -0.69 | 5.53 – 17.46 | -0.87 – -0.50 | -7.25 | 0.00 |
| timepoint [moderate] | 7.65 | 3.16 | 0.24 | 1.45 – 13.84 | 0.05 – 0.43 | 2.42 | 0.02 |
| timepoint [peak] | 24.97 | 3.16 | 0.78 | 18.78 – 31.17 | 0.59 – 0.97 | 7.90 | 0.00 |
| timepoint [removal] | 46.88 | 3.16 | 1.46 | 40.69 – 53.08 | 1.27 – 1.66 | 14.84 | 0.00 |
| Group [YesSA] | 0.56 | 5.77 | 0.02 | -10.74 – 11.87 | -0.33 – 0.37 | 0.10 | 0.92 |
| timepoint [moderate] \* Group [YesSA] | 3.47 | 6.01 | 0.11 | -8.30 – 15.25 | -0.26 – 0.48 | 0.58 | 0.56 |
| timepoint [peak] \* Group [YesSA] | 6.24 | 6.01 | 0.19 | -5.53 – 18.02 | -0.17 – 0.56 | 1.04 | 0.30 |
| timepoint [removal] \* Group [YesSA] | 16.69 | 5.99 | 0.52 | 4.95 – 28.42 | 0.15 – 0.89 | 2.79 | 0.01 |
| Random Effects | | | | | | | |
| σ2 | 339.54 | | | | | | || τ00 id | 289.85 | | | | | | || ICC | 0.46 | | | | | | || N id | 95 | | | | | | || Observations | 377 | | | | | | |
| Marginal R2 / Conditional R2 | 0.395 / 0.674 | | | | | | |

```
r2beta(cp_mod, method = "kr")
```

```
##            Effect   Rsq upper.CL lower.CL
## 1           Model 0.593    0.653    0.536
## 2       timepoint 0.589    0.650    0.526
## 4 timepoint:Group 0.030    0.089    0.008
## 3           Group 0.027    0.125    0.000
```

---

### Plot of Cold Pressor Results (Figure 2)

```
cp %>% 
    mutate(Group = factor(Group, 
    levels=c("YesSA","NoSA"))) %>% 
  ggplot() + theme_classic() +
  aes(x=timepoint, linetype=Group, group=Group, y=value) + 
  stat_summary(fun.y = mean, geom="point") +
  stat_summary(fun.y = mean, geom="line") +
  stat_summary(fun.data = mean_se, geom = "errorbar", width=.2) +
  scale_x_discrete(" ",
                   labels=c("Mild Pain", "Moderate Pain", "Peak Pain", "Hand Removal")) +
  scale_y_continuous("Time (seconds)", breaks=c(0,15,30,45,60,75,90)) +
  scale_linetype_manual(" ", values=c("solid", "dotted"), labels=c("Attempters", "Non-Attempters")) +
  theme(axis.title.y = element_text(size=14),
        axis.text.y = element_text(size=12),
        axis.text.x = element_text(size=12))
```

---

### Comparison of cold pressor VAS ratings and plots

```
wilcox.test(my.data$cp_stressful ~ my.data$Group, paired = FALSE, correct = FALSE)
```

```
## 
##  Wilcoxon rank sum test
## 
## data:  my.data$cp_stressful by my.data$Group
## W = 1095, p-value = 0.1439
## alternative hypothesis: true location shift is not equal to 0
```

```
wilcoxonR(x = my.data$cp_stressful, 
          g = my.data$Group)
```

```
##     r 
## 0.145
```

```
wilcox.test(my.data$cp_difficulty ~ my.data$Group, paired = FALSE, correct = FALSE)
```

```
## 
##  Wilcoxon rank sum test
## 
## data:  my.data$cp_difficulty by my.data$Group
## W = 1123, p-value = 0.09013
## alternative hypothesis: true location shift is not equal to 0
```

```
wilcoxonR(x = my.data$cp_difficulty, 
          g = my.data$Group)
```

```
##     r 
## 0.167
```

```
wilcox.test(my.data$cp_unpleasantness ~ my.data$Group, paired = FALSE, correct = FALSE)
```

```
## 
##  Wilcoxon rank sum test
## 
## data:  my.data$cp_unpleasantness by my.data$Group
## W = 1107, p-value = 0.1167
## alternative hypothesis: true location shift is not equal to 0
```

```
wilcoxonR(x = my.data$cp_unpleasantness, 
          g = my.data$Group)
```

```
##     r 
## 0.155
```

```
wilcox.test(my.data$cp_peak_pain_corrected ~ my.data$Group, paired = FALSE, correct = FALSE)
```

```
## 
##  Wilcoxon rank sum test
## 
## data:  my.data$cp_peak_pain_corrected by my.data$Group
## W = 1066.5, p-value = 0.1218
## alternative hypothesis: true location shift is not equal to 0
```

```
wilcoxonR(x = my.data$cp_peak_pain_corrected, 
          g = my.data$Group)
```

```
##     r 
## 0.153
```

```
cprat.p = c(.1439, .0901, .1167, .1218)
p.adjust(cprat.p, method = "BH")
```

```
## [1] 0.1439 0.1439 0.1439 0.1439
```

```
#adjusted p-values = .144, .144, .144, .144
```

---

### Plot of mean VAS ratings by group (Figure 2)

```
my.data$cp_peak_pain100 = my.data$cp_peak_pain_corrected*100 #multiply by 100 to get pain rating on same scale as unpleasantness, difficulty, and stress
painvars = my.data %>% dplyr::select("id", 
                               "Group", 
                               "cp_peak_pain100", 
                               "cp_unpleasantness", 
                               "cp_difficulty", 
                               "cp_stressful")
pain = gather(painvars,
               value = "value",
               key = "var",
               cp_peak_pain100, cp_unpleasantness, cp_difficulty, cp_stressful)

pain = pain %>% mutate(Group = factor(Group, 
    levels=c("YesSA","NoSA"))) %>% 
  mutate(var = factor(var,
    levels=c("cp_unpleasantness", "cp_peak_pain100", "cp_difficulty", "cp_stressful")))
  

ggplot(pain, aes(x=var, y=value, fill=Group)) + theme_classic() +
  stat_summary(fun.y = mean, geom="bar", position ="dodge", color = "black") +
  stat_summary(fun.data = mean_se, geom = "errorbar", width=.2, position=position_dodge(.9)) +
  scale_x_discrete(" ",
                   labels=c("Unpleasantness","Pain","Difficulty","Stress")) +
  scale_y_continuous("VAS Ratings") +
    scale_fill_manual(values=c("#3182bd","#deebf7"), label=c("Attempters","Non-Attempters")) +
  ggtitle("Cold Pressor Challenge Ratings") +
  theme(axis.title.y = element_text(size=14),
        axis.text.y = element_text(size=12),
        axis.text.x = element_text(size=12))
```

---

## Heartbeat Accuracy Analysis - Includes guess condition

#### Results reported on page 14 of manusript

---

```
acc = my.data %>% dplyr::select ("id",
                                 "Group",
                                 "t0_counting_accuracy",
                                 "t2_counting_accuracy",
                                 "t3_counting_accuracy")

acc <- gather(acc,
               value = "value",
               key = "var",
              t0_counting_accuracy, t2_counting_accuracy, t3_counting_accuracy)   

#LME for heartbeat tapping accuracy by group and condition
heart_acc_mod <- lmer(value ~ var*Group + (1|id), data = acc, na.action = na.exclude)
anova(heart_acc_mod, ddf = "Kenward-Roger", type = "marginal")
```

```
## Marginal Analysis of Variance Table with Kenward-Roger's method
##            Sum Sq Mean Sq NumDF  DenDF F value    Pr(>F)    
## var       1.94938 0.97469     2 195.08 12.7153 6.446e-06 ***
## Group     0.03604 0.03604     1 288.10  0.4702    0.4935    
## var:Group 0.22504 0.11252     2 195.92  1.4679    0.2329    
## ---
## Signif. codes:  0 '***' 0.001 '**' 0.01 '*' 0.05 '.' 0.1 ' ' 1
```

```
summary(heart_acc_mod, ddf = "Kenward-Roger")
```

```
## Linear mixed model fit by REML. t-tests use Kenward-Roger's method [
## lmerModLmerTest]
## Formula: value ~ var * Group + (1 | id)
##    Data: acc
## 
## REML criterion at convergence: 128
## 
## Scaled residuals: 
##     Min      1Q  Median      3Q     Max 
## -4.5465 -0.6885  0.0257  0.7030  2.2414 
## 
## Random effects:
##  Groups   Name        Variance Std.Dev.
##  id       (Intercept) 0.007803 0.08834 
##  Residual             0.076655 0.27687 
## Number of obs: 299, groups:  id, 100
## 
## Fixed effects:
##                                     Estimate Std. Error        df t value
## (Intercept)                          0.67665    0.03524 288.09847  19.200
## vart2_counting_accuracy             -0.22035    0.04748 195.07629  -4.641
## vart3_counting_accuracy             -0.19133    0.04748 195.07629  -4.030
## GroupYesSA                          -0.04272    0.06230 288.09847  -0.686
## vart2_counting_accuracy:GroupYesSA  -0.13267    0.08394 195.07629  -1.581
## vart3_counting_accuracy:GroupYesSA  -0.01787    0.08444 196.35117  -0.212
##                                    Pr(>|t|)    
## (Intercept)                         < 2e-16 ***
## vart2_counting_accuracy            6.36e-06 ***
## vart3_counting_accuracy            8.00e-05 ***
## GroupYesSA                            0.493    
## vart2_counting_accuracy:GroupYesSA    0.116    
## vart3_counting_accuracy:GroupYesSA    0.833    
## ---
## Signif. codes:  0 '***' 0.001 '**' 0.01 '*' 0.05 '.' 0.1 ' ' 1
## 
## Correlation of Fixed Effects:
##             (Intr) vrt2__ vrt3__ GrpYSA v2__:G
## vrt2_cntng_ -0.674                            
## vrt3_cntng_ -0.674  0.500                     
## GroupYesSA  -0.566  0.381  0.381              
## vrt2__:GYSA  0.381 -0.566 -0.283 -0.674       
## vrt3__:GYSA  0.379 -0.281 -0.562 -0.670  0.497
```

```
sjPlot::tab_model(heart_acc_mod, show.std = TRUE, show.se=TRUE, show.stat = TRUE)
```

|  | value | | | | | | |
| --- | --- | --- | --- | --- | --- | --- | --- |
| Predictors | Estimates | std. Error | std. Beta | CI | standardized CI | Statistic | p |
| (Intercept) | 0.68 | 0.04 | 0.53 | 0.61 – 0.75 | 0.31 – 0.75 | 4.73 | 0.00 |
| var [t2\_counting\_accuracy] | -0.22 | 0.05 | -0.70 | -0.31 – -0.13 | -1.00 – -0.41 | -4.64 | 0.00 |
| var [t3\_counting\_accuracy] | -0.19 | 0.05 | -0.61 | -0.28 – -0.10 | -0.91 – -0.31 | -4.03 | 0.00 |
| Group [YesSA] | -0.04 | 0.06 | -0.14 | -0.16 – 0.08 | -0.53 – 0.25 | -0.69 | 0.49 |
| var [t2\_counting\_accuracy] \* Group [YesSA] | -0.13 | 0.08 | -0.42 | -0.30 – 0.03 | -0.95 – 0.10 | -1.58 | 0.11 |
| var [t3\_counting\_accuracy] \* Group [YesSA] | -0.02 | 0.08 | -0.06 | -0.18 – 0.15 | -0.59 – 0.47 | -0.21 | 0.83 |
| Random Effects | | | | | | | |
| σ2 | 0.08 | | | | | | || τ00 id | 0.01 | | | | | | || ICC | 0.09 | | | | | | || N id | 100 | | | | | | || Observations | 299 | | | | | | |
| Marginal R2 / Conditional R2 | 0.152 / 0.231 | | | | | | |

```
r2beta(heart_acc_mod, method = 'kr')
```

```
##      Effect   Rsq upper.CL lower.CL
## 1     Model 0.201    0.305    0.130
## 2       var 0.200    0.304    0.116
## 3     Group 0.054    0.166    0.002
## 4 var:Group 0.015    0.072    0.001
```

---

## Heartbeat Accuracy Analysis - Guessing condition omitted

#### Results reported on page 14 of manuscript and depicted in Figure 3

---

```
acc_noguess = my.data %>% dplyr::select ("id",
                                 "Group",
                                 "t2_counting_accuracy",
                                 "t3_counting_accuracy")

acc_noguess <- gather(acc_noguess,
               value = "value",
               key = "var",
               t2_counting_accuracy, t3_counting_accuracy) 

#LME for heartbeat tapping accuracy by group and condition
heart_noguess_mod <- lmer(value ~ var*Group + (1|id), data = acc_noguess, na.action = na.exclude)
anova(heart_noguess_mod, ddf = "Kenward-Roger", type = "marginal")
```

```
## Marginal Analysis of Variance Table with Kenward-Roger's method
##             Sum Sq  Mean Sq NumDF   DenDF F value   Pr(>F)   
## var       0.028622 0.028622     1  97.041  0.9086 0.342846   
## Group     0.272094 0.272094     1 144.466  8.6379 0.003834 **
## var:Group 0.137549 0.137549     1  97.722  4.3666 0.039246 * 
## ---
## Signif. codes:  0 '***' 0.001 '**' 0.01 '*' 0.05 '.' 0.1 ' ' 1
```

```
summary(heart_noguess_mod, ddf = "Kenward-Roger")
```

```
## Linear mixed model fit by REML. t-tests use Kenward-Roger's method [
## lmerModLmerTest]
## Formula: value ~ var * Group + (1 | id)
##    Data: acc_noguess
## 
## REML criterion at convergence: 27.9
## 
## Scaled residuals: 
##      Min       1Q   Median       3Q      Max 
## -1.97019 -0.55569 -0.09871  0.55057  2.23555 
## 
## Random effects:
##  Groups   Name        Variance Std.Dev.
##  id       (Intercept) 0.04599  0.2145  
##  Residual             0.03150  0.1775  
## Number of obs: 199, groups:  id, 100
## 
## Fixed effects:
##                                     Estimate Std. Error        df t value
## (Intercept)                          0.45630    0.03376 144.46557  13.517
## vart3_counting_accuracy              0.02901    0.03044  97.04104   0.953
## GroupYesSA                          -0.17539    0.05968 144.46557  -2.939
## vart3_counting_accuracy:GroupYesSA   0.11343    0.05428  97.72213   2.090
##                                    Pr(>|t|)    
## (Intercept)                         < 2e-16 ***
## vart3_counting_accuracy             0.34285    
## GroupYesSA                          0.00383 ** 
## vart3_counting_accuracy:GroupYesSA  0.03925 *  
## ---
## Signif. codes:  0 '***' 0.001 '**' 0.01 '*' 0.05 '.' 0.1 ' ' 1
## 
## Correlation of Fixed Effects:
##             (Intr) vrt3__ GrpYSA
## vrt3_cntng_ -0.451              
## GroupYesSA  -0.566  0.255       
## vrt3__:GYSA  0.253 -0.561 -0.447
```

```
sjPlot::tab_model(heart_noguess_mod, show.std = TRUE, show.se=TRUE, show.stat = TRUE)
```

|  | value | | | | | | |
| --- | --- | --- | --- | --- | --- | --- | --- |
| Predictors | Estimates | std. Error | std. Beta | CI | standardized CI | Statistic | p |
| (Intercept) | 0.46 | 0.03 | 0.08 | 0.39 – 0.52 | -0.15 – 0.31 | 0.68 | 0.49 |
| var [t3\_counting\_accuracy] | 0.03 | 0.03 | 0.10 | -0.03 – 0.09 | -0.11 – 0.31 | 0.95 | 0.34 |
| Group [YesSA] | -0.18 | 0.06 | -0.62 | -0.29 – -0.06 | -1.03 – -0.20 | -2.94 | 0.00 |
| var [t3\_counting\_accuracy] \* Group [YesSA] | 0.11 | 0.05 | 0.40 | 0.01 – 0.22 | 0.02 – 0.77 | 2.09 | 0.04 |
| Random Effects | | | | | | | |
| σ2 | 0.03 | | | | | | || τ00 id | 0.05 | | | | | | || ICC | 0.59 | | | | | | || N id | 100 | | | | | | || Observations | 199 | | | | | | |
| Marginal R2 / Conditional R2 | 0.059 / 0.618 | | | | | | |

```
r2beta(heart_noguess_mod, method = 'kr')
```

```
##      Effect   Rsq upper.CL lower.CL
## 1     Model 0.108    0.232    0.040
## 2       var 0.093    0.221    0.014
## 3     Group 0.048    0.157    0.001
## 4 var:Group 0.043    0.149    0.001
```

---

### Plot for heartbeat perception accuracy means by group (Figure 3)

```
my.data %>% 
      mutate(Group = factor(Group, 
    levels=c("YesSA","NoSA"))) %>% 
  ggplot() + theme_classic() +
  aes(x=Group,y=mean_t2t3acc,fill=Group) +  
  geom_dotplot(binaxis='y', stackdir='center', alpha =.8, position = "dodge") +
  geom_crossbar(stat="summary", fun.y=mean, fun.ymax=mean, fun.ymin=mean, fatten=1.5, width = 0.4)+ 
    scale_fill_manual(values=c("#3182bd","#deebf7"), label=c("Attempters","Non-Attempters")) +
  scale_x_discrete(" ",
                   labels=c("Attempters","Non-Attempters")) +
  scale_y_continuous("Heartbeat Perception Accuracy", breaks=c(0.0, 0.25,0.50,0.75,1.00)) +
  coord_cartesian(ylim=c(0,1)) +
  theme(axis.title.y = element_text(size=14),
        axis.text.y = element_text(size=14),
        axis.text.x = element_text(size=14))
```

---

### Comparison of VAS Ratings for heartbeat perception task

```
wilcox.test(my.data$mean_t2t3conf ~ my.data$Group, paired = FALSE, correct = FALSE)
```

```
## 
##  Wilcoxon rank sum test
## 
## data:  my.data$mean_t2t3conf by my.data$Group
## W = 1153.5, p-value = 0.4528
## alternative hypothesis: true location shift is not equal to 0
```

```
wilcoxonR(x = my.data$mean_t2t3conf, 
          g = my.data$Group)
```

```
##      r 
## 0.0744
```

```
wilcox.test(my.data$mean_t2t3diff ~ my.data$Group, paired = FALSE, correct = FALSE)
```

```
## 
##  Wilcoxon rank sum test
## 
## data:  my.data$mean_t2t3diff by my.data$Group
## W = 1014.5, p-value = 0.7657
## alternative hypothesis: true location shift is not equal to 0
```

```
wilcoxonR(x = my.data$mean_t2t3diff, 
          g = my.data$Group)
```

```
##       r 
## -0.0295
```

```
wilcox.test(my.data$mean_t2t3intens ~ my.data$Group, paired = FALSE, correct = FALSE)
```

```
## 
##  Wilcoxon rank sum test
## 
## data:  my.data$mean_t2t3intens by my.data$Group
## W = 1345, p-value = 0.0281
## alternative hypothesis: true location shift is not equal to 0
```

```
wilcoxonR(x = my.data$mean_t2t3intens, 
          g = my.data$Group)
```

```
##     r 
## 0.218
```

```
#Benjamini correction for group contrast across difficulty, confidence, and intensity
hbrat.p = c(.453, .767, .028)
p.adjust(hbrat.p, method = "BH")
```

```
## [1] 0.6795 0.7670 0.0840
```

---

### Plot for heartbeat VAS rating means by group (Figure 3)

```
hbpVAS = my.data %>% 
  dplyr::select("id", "Group", "mean_t2t3conf", "mean_t2t3diff", "mean_t2t3intens") %>% 
      mutate(Group = factor(Group, levels=c("YesSA","NoSA"))) 

  
hbpVAS <- gather(hbpVAS,
                      value = "value",
                      key = "var",
                      mean_t2t3conf, mean_t2t3diff, mean_t2t3intens)

ggplot(hbpVAS, aes(x=var, y=value, fill=Group)) + theme_classic()+
  stat_summary(fun.y = mean, geom="bar", position ="dodge", color = "black") +
  stat_summary(fun.data = mean_se, geom = "errorbar", width=.2, position=position_dodge(.9)) +
  scale_x_discrete(" ",
                   labels=c("Confidence", "Difficulty", "Intensity")) +
  scale_y_continuous("VAS Ratings") +
    scale_fill_manual(values=c("#3182bd","#deebf7"), label=c("Attempters","Non-Attempters")) +
  ggtitle("Heartbeat Perception Task Ratings") +
  coord_cartesian(ylim=c(0,65)) +
  theme(axis.title.y = element_text(size=14),
        axis.text.y = element_text(size=12),
        axis.text.x = element_text(size=12))
```

---

## Plot of beta coefficient means by group for Figure 4

```
IA_betas = my.data %>% dplyr::select("id", "Group", "beta_postIns_HvT", "beta_dmIns_HvT") %>% 
        mutate(Group = factor(Group, levels=c("YesSA","NoSA"))) 


IA_betas <- gather(IA_betas,
                   value = "value",
                   key = "var",
                   beta_postIns_HvT, beta_dmIns_HvT)


ggplot(IA_betas, aes(x=var, y=value, fill=Group)) + theme_classic() +
  stat_summary(fun.y = mean, geom="bar", position ="dodge", color = "black") +
  stat_summary(fun.data = mean_se, geom = "errorbar", width=.2, position=position_dodge(.9)) +
  scale_x_discrete(" ",
                   labels=c("Mid Insula", "Posterior Insula")) +
  scale_y_continuous("% BOLD Signal Change") +
  scale_fill_manual(values=c("#3182bd","#deebf7"), label=c("Attempters","Non-Attempters")) +
  coord_cartesian(ylim=c(-0.15,0.15)) +
  theme(axis.title.y = element_text(size=14),
        axis.text.y = element_text(size=12),
        axis.text.x = element_text(size=12))
```
