# Supplementary material for: Diminished responses to bodily threat and blunted interoception in suicide attempters
Source: eLife. 2020 Apr 7;9:e51593. doi: 10.7554/eLife.51593 (PMC7138608; doi:10.7554/eLife.51593)
Supplement: Supplementary file 1. [file elife-51593-supp1.docx]

**Supplemental Table.** Output of linear mixed effects models for breath hold duration, CO_2_, and O_2_.

| **Predictors** ^a^ | **Estimate (SE)** ^b^ | | **Std. Estimate (SE)** ^c^ | **df** | **t-value** | **p-value** | |
| --- | --- | --- | --- | --- | --- | --- | --- |
| ***Duration~ Group x Trial + (1\|id)*** | |  |  |  |  |  | |
| **Fixed Effects** |  | |  |  |  |  | |
| Intercept | 42.98 (2.78) | | -- | 120.32 | 15.46 | <0.001 | |
| Attempter | 10.45 (4.94) | | 0.21 (0.10) | 121.84 | 2.12 | 0.036 | |
| Trial 2 | 8.07 (1.80) | | 0.17 (0.04) | 97.01 | 4.50 | <0.001 | |
| Trial 2 x Attempter | -1.65 (3.21) | | -0.03 (0.05) | 97.40 | -0.52 | 0.606 | |
| **Random Effects** ^d^ |  | |  |  | |  |  |
| σ^2^  τ_00_ _id_  ICC _id_  **Marginal R^2^ / Conditional R^2^** | 109.84  415.97  0.79  0.06/0.80 | |  |  | |  |  |
| ***CO_2_ ~ Group x Trial + (1\|id)*** |  | |  |  |  |  | |
| **Fixed Effects** |  | |  |  |  |  | |
| Intercept | 5.82 (0.08) | | -- | 118.41 | 71.66 | <0.001 | |
| Attempter | 0.36 (0.15) | | 0.24 (0.10) | 120.33 | 2.35 | 0.019 | |
| Trial 2 | 0.04 (0.06) | | 0.03 (0.04) | 91.04 | 0.75 | 0.454 | |
| Attempter x Trial 2 | -0.09 (0.11) | | -0.05 (0.05) | 92.70 | -0.87 | 0.386 | |
| **Random Effects** |  | |  |  | |  |  |
| σ^2^  τ_00_ _id_  ICC _id_  **Marginal R^2^ / Conditional R^2^** | 0.11  0.34  0.76  0.04/0.77 | |  |  | |  |  |
| ***O_2_ ~ Group x Trial + (1\|id)*** |  | |  |  |  |  | |
| **Fixed Effects** |  | |  |  |  |  | |
| Intercept | 13.27 (0.20) | | -- | 129.96 | 65.04 | <0.001 | |
| Attempter | -0.85 (0.38) | | -0.22 (0.10) | 132.37 | -2.24 | 0.025 | |
| Trial 2 | -0.42 (0.17) | | -0.12 (0.05) | 91.09 | -2.48 | 0.013 | |
| Attempter x Trial 2 | 0.39 (0.32) | | 0.08 (0.07) | 93.31 | 1.20 | 0.228 | |
| **Random Effects** |  | |  |  | |  |  |
| σ^2^  τ_00_ _id_  ICC _id_  **Marginal R^2^ / Conditional R^2^** | 0.97  1.86  0.66  0.04/0.67 | |  |  | |  | |

^a^ The factor “group” contained two levels: non-attempter and suicide attempter. The “trial” factor also contained two levels: trial 1 and trial 2. The intercept was set to non-attempter, trial 1.

^b^ “SE” = standard error; ^c^ “Std. Estimate” refers to the standardized regression coefficient.

^d^ σ^2^ = variance within groups, τ_00_ _id_ = variance between groups, ICC _id_ = intraclass correlation
