# Supplementary material for: Diminished responses to bodily threat and blunted interoception in suicide attempters
Source: eLife. 2020 Apr 7;9:e51593. doi: 10.7554/eLife.51593 (PMC7138608; doi:10.7554/eLife.51593)
Supplement: Supplementary file 2. [file elife-51593-supp2.docx]

**Supplement Table.** Output of linear mixed effects model for cold pressor challenge.

| **Predictors** ^a^ | **Estimate (SE)** ^b^ | **Std. Estimate (SE)** ^c^ | **df** | **t-value** | **p-value** |
| --- | --- | --- | --- | --- | --- |
| ***Duration ~ Timepoint x Group + (1\|id)*** | | | | | |
| **Fixed Effects** |  |  |  |  |  |
| Intercept | 11.49 (3.04) | -- | 225.90 | 3.78 | <0.001 |
| Moderate Pain | 7.65 (3.16) | 0.10 (0.04) | 276.16 | 2.42 | 0.016 |
| Peak Pain | 24.97 (3.16) | 0.34 (0.04) | 276.16 | 7.90 | <0.001 |
| Hand Removal | 46.88 (3.16) | 0.64 (0.04) | 276.16 | 14.84 | <0.001 |
| Group (Attempter) | 0.56 (5.77) | 0.01 (0.08) | 229.98 | 0.10 | 0.922 |
| Moderate x Attempter | 3.47 (6.01) | 0.03 (0.05) | 276.16 | 0.58 | 0.563 |
| Peak x Attempter | 6.24 (6.01) | 0.05 (0.05) | 276.16 | 1.04 | 0.300 |
| Hand Removal x Attempter | 16.69 (5.99) | 0.13 (0.05) | 276.56 | 2.79 | 0.005 |
| **Random Effects**^d^ |  |  |  |  |  |
| σ^2^ | 339.54 |  |  |  |  |
| τ_00_ _id_ | 28.85 |  |  |  |  |
| ICC _id_ | 0.46 |  |  |  |  |
| **Marginal R^2^ / Conditional R^2^** | 0.40/0.67 |  |  |  |  |

^a^ The “timepoint” factor contained four levels, including the time at which participants provided ratings of mild pain (25/100), moderate pain (50/100), peak pain (100/100 or maximum value), and the time at which they removed their hand from the cold-pressor. The “group” factor had two levels: non-attempter and suicide attempter. The intercept was set to non-attempter, mild pain.

^b^ “SE” = standard error; ^c^ “Std. Estimate” refers to the standardized regression coefficient.

^d^ σ^2^ = variance within groups, τ_00_ _id_ = variance between groups, ICC _id_ = intraclass correlation
