# Supplementary material for: Diminished responses to bodily threat and blunted interoception in suicide attempters
Source: eLife. 2020 Apr 7;9:e51593. doi: 10.7554/eLife.51593 (PMC7138608; doi:10.7554/eLife.51593)
Supplement: Supplementary file 3. [file elife-51593-supp3.docx]

**Supplemental Table.** Output of linear mixed effects model for heartbeat perception models.

^a^ In the first model, the factor “condition” contained three levels: *Guess:* providing the best guess of one’s own heartbeat, *No Guess:* sensing one’s heartbeat without guessing, and *Perturbation:* sensing one’s heartbeat during interoceptive perturbation (i.e., while holding one’s breath). In the second model, the factor “condition” contained only the *No Guess* and *Perturbation* levels. In both models, the “group” factor contained two levels: non-attempter and suicide attempter. In the first model, the intercept was set to *No Guess, Non-Attempter*. In the second model, the intercept was set to *Guess*, *Non-Attempter.*

^b^ “SE” = standard error; ^c^ “Std. Estimate” refers to the standardized regression coefficient.

^d^ σ^2^ = variance within groups, τ_00_ _id_ = variance between groups, ICC _id_ = intraclass correlation

| **Predictors** ^a^ | **Estimate (SE)** ^b^ | **Std. Estimate (SE)** ^c^ | **df** | **t-value** | **p-value** |  |
| --- | --- | --- | --- | --- | --- | --- |
| ***Interoceptive Accuracy ~ Condition [Guess, No guess, Perturbation] x Group + (1\|ID)*** | | | | | | |
| Intercept | 0.68 (0.04) | -- | 288.08 | 19.20 | <0.001 |  |
| No Guess Trial | -0.22 (0.05) | -0.33 (0.07) | 195.08 | -4.64 | <0.001 |  |
| Perturbation | -0.19 (0.05) | -0.29 (0.07) | 195.08 | -4.03 | <0.001 |  |
| Attempter | -0.04 (0.06) | -0.06 (0.09) | 288.09 | -0.69 | 0.493 |  |
| Attempter x No Guess Trial | -0.13 (0.08) | -0.13 (0.08) | 195.08 | -1.59 | 0.116 |  |
| Attempter x Perturbation Trial | -0.02 (0.08) | -0.02(0.08) | 196.35 | -0.21 | 0.833 |  |
| **Random Effects** ^d^ |  |  |  |  |  |  |
| σ^2^ | 0.08 |  |  |  |  |  |
| τ_00_ _id_  ICC _id_  **Marginal R^2^ / Conditional R^2^** | 0.01  0.09  0.15/0.23 |  |  |  |  |  |
| ***Interoceptive Accuracy ~ Condition [No Guess, Perturbation] x Group + (1\|ID)*** | | | | | |  |
| Intercept | 0.46 (0.03) | -- | 144.47 | 13.52 | <0.001 |  |
| Perturbation | 0.03 (0.05) | -0.05 (0.05) | 97.04 | 0.95 | 0.343 |  |
| Attempter | -0.18 (0.06) | -0.29 (0.10) | 144.47 | -2.94 | 0.004 |  |
| Attempter x Perturbation | 0.11 (0.08) | 0.14 (0.07 | 97.72 | 2.09 | 0.039 |  |
| **Random Effects** ^d^ |  |  |  |  |  |  |
| σ^2^ | 0.08 |  |  |  |  |  |
| τ_00_ _id_  ICC _id_  **Marginal R^2^ / Conditional R^2^** | 0.01  0.09  0.15/0.23 |  |  |  |  |  |
|  |  |  |  |  |  |  |
