# Supplementary material for: Diminished responses to bodily threat and blunted interoception in suicide attempters
Source: eLife. 2020 Apr 7;9:e51593. doi: 10.7554/eLife.51593 (PMC7138608; doi:10.7554/eLife.51593)
Supplement: Transparent reporting form [file elife-51593-transrepform.pdf]

eLIFE
1st Floor
24 Hills Road
Cambridge CB2 1JP, UK
P 01223 855340
W elifesciences.org
T @elife

# *eLife's* transparent reporting form

We encourage authors to provide detailed information *within their submission* to facilitate the interpretation and replication of experiments. Authors can upload supporting documentation to indicate the use of appropriate reporting guidelines for health-related research (see EQUATOR Network), life science research (see the BioSharing Information Resource), or the ARRIVE guidelines for reporting work involving animal research. Where applicable, authors should refer to any relevant reporting standards documents in this form.

If you have any questions, please consult our Journal Policies and/or contact us: editorial@elifesciences.org.

**Sample-size estimation**

- You should state whether an appropriate sample size was computed when the study was being designed
- You should state the statistical method of sample size computation and any required assumptions
- If no explicit power analysis was used, you should describe how you decided what sample (replicate) size (number) to use

Please outline where this information can be found within the submission (e.g., sections or figure legends), or explain why this information doesn't apply to your submission:

We report on a retrospective analysis of a sub-sample from a pre-existing dataset. We included all available subjects with a history of a suicide attempt. Therefore, we did not conduct power analyses to determine sample size. Detailed information about our sample is provided under "Participants" (pp. 15 in main manuscript). For readers who may be interested, additional information regarding our sample characteristics and matching procedures is also provided in Appendix 1.

**Replicates**

- You should report how often each experiment was performed
- You should include a definition of biological versus technical replication
- The data obtained should be provided and sufficient information should be provided to indicate the number of independent biological and/or technical replicates
- If you encountered any outliers, you should describe how these were handled
- Criteria for exclusion/inclusion of data should be clearly stated
- High-throughput sequence data should be uploaded before submission, with a private link for reviewers provided (these are available from both GEO and ArrayExpress)

Please outline where this information can be found within the submission (e.g., sections or figure legends), or explain why this information doesn't apply to your submission:

The experiments described were performed once using human research subjects. We did not encounter outliers. Details regarding inclusion and exclusion of participants, missing data, omission of data points due to poor quality (e.g., poor anatomical alignment in fMRI data) are provided in Appendix 1(page 2) in the paragraph labeled "Missing Data" and referenced in the main text (page 4).

eLife Sciences Publications, Ltd is a limited liability non-profit non-stock corporation incorporated in the State of Delaware, USA, with company number 5030732, and is registered in the UK with company number FC030576 and branch number BR015634 at the address 1st Floor, 24 Hills Road, Cambridge CB2 1JP | August 2014

**Statistical reporting**

- Statistical analysis methods should be described and justified
- Raw data should be presented in figures whenever informative to do so (typically when N per group is less than 10)
- For each experiment, you should identify the statistical tests used, exact values of N, definitions of center, methods of multiple test correction, and dispersion and precision measures (e.g., mean, median, SD, SEM, confidence intervals; and, for the major substantive results, a measure of effect size (e.g., Pearson's r, Cohen's d)
- Report exact p-values wherever possible alongside the summary statistics and 95% confidence intervals. These should be reported for all key questions and not only when the p-value is less than 0.05.

Please outline where this information can be found within the submission (e.g., sections or figure legends), or explain why this information doesn't apply to your submission:

Regarding descriptive data, means and standard deviations for clinical variables are presented in Table 1. Each figure (pp. 31-34) depicts means and standard errors for behavioral, neuroimaging, and VAS rating measures. Where we did not include standard errors, we used dot plots to provide the audience with an informative visualization of the dispersion within and between each group (Figures 1 and 3). Information regarding sample size and statistical analyses used can be found in the Methods section of our manuscript (pp. 15-22). Degrees of freedom for each statistical test are also reported throughout the Results section of the main manuscript (pp. 4-9). For all statistical tests, summary statistics, exact p-values are provided. Measures of effect size are provided for each fixed effect (see Results section, pp. 15-21). Exact model output is provided in Supplementary Files 1-3.

(For large datasets, or papers with a very large number of statistical tests, you may upload a single table file with tests, Ns, etc., with reference to sections in the manuscript.)

**Group allocation**

- Indicate how samples were allocated into experimental groups (in the case of clinical studies, please specify allocation to treatment method); if randomization was used, please also state if restricted randomization was applied
- Indicate if masking was used during group allocation, data collection and/or data analysis

Please outline where this information can be found within the submission (e.g., sections or figure legends), or explain why this information doesn't apply to your submission:

This information can be found under "Participants" in the Materials and Methods section (p. 15).

**Additional data files ("source data")**

- We encourage you to upload relevant additional data files, such as numerical data that are represented as a graph in a figure, or as a summary table
- Where provided, these should be in the most useful format, and they can be uploaded as "Source data" files linked to a main figure or table
- Include model definition files including the full list of parameters used
- Include code used for data analysis (e.g., R, MatLab)
- Avoid stating that data files are "available upon request"

Please indicate the figures or tables for which source data files have been provided:

De-identified processed source data for each Figure has been provided. Information regarding the clinical characteristics of our sample (as described in Table 1) has not been shared to protect the privacy and confidentiality of our research participants. R code for data analysis and figure creation has also been provided.
